# 22% Efficiency Inverted Perovskite Photovoltaic Cell Using Cation‐Doped Brookite TiO_2_ Top Buffer

**DOI:** 10.1002/advs.202001285

**Published:** 2020-07-02

**Authors:** Xiaowen Hu, Chang Liu, Zhiyong Zhang, Xiao‐Fang Jiang, Juan Garcia, Colton Sheehan, Lingling Shui, Shashank Priya, Guofu Zhou, Sen Zhang, Kai Wang

**Affiliations:** ^1^ Guangdong Provincial Key Laboratory of Optical Information Materials and Technology & Institute of Electronic Paper Displays South China Academy of Advanced Optoelectronics South China Normal University Guangzhou 510006 China; ^2^ SCNU‐TUE Joint Lab of Device Integrated Responsive Materials (DIRM) National Center for International Research on Green Optoelectronics South China Normal University Guangzhou 510006 China; ^3^ Department of Chemistry University of Virginia Charlottesville VA 22904 USA; ^4^ Material Research Institute Pennsylvania State University University Park PA 16802 USA

**Keywords:** durability, high efficiency, interface engineering, inverted perovskite solar cells

## Abstract

Simultaneously achieving high efficiency and high durability in perovskite solar cells is a critical step toward the commercialization of this technology. Inverted perovskite photovoltaic (IP‐PV) cells incorporating robust and low levelized‐cost‐of‐energy (LCOE) buffer layers are supposed to be a promising solution to this target. However, insufficient inventory of materials for back‐electrode buffers substantially limits the development of IP‐PV. Herein, a composite consisting of 1D cation‐doped TiO_2_ brookite nanorod (NR) embedded by 0D fullerene is investigated as a top modification buffer for IP‐PV. The cathode buffer is constructed by introducing fullerene to fill the interstitial space of the TiO_2_ NR matrix. Meanwhile, cations of transition metal Co or Fe are doped into the TiO_2_ NR to further tune the electronic property. Such a top buffer exhibits multifold advantages, including improved film uniformity, enhanced electron extraction and transfer ability, better energy level matching with perovskite, and stronger moisture resistance. Correspondingly, the resultant IP‐PV displays an efficiency exceeding 22% with a 22‐fold prolonged working lifetime. The strategy not only provides an essential addition to the material inventory for top electron buffers by introducing the 0D:1D composite concept, but also opens a new avenue to optimize perovskite PVs with desirable properties.

## Graphical abstract

A composite consisting of 1D cation‐doped TiO_2_ brookite nanorod embedded by 0D fullerene is investigated as a top modification buffer for inverted perovskite photovoltaic (IP‐PV) cells. The resultant IP‐PV displays an efficiency exceeding 22% with a favorable stability. This work opens up more opportunities in expanding the material inventory for charge transfer layer in perovskite solar cells development and application.

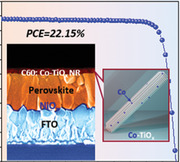

Organic–inorganic halide perovskites have captured growing attention in various energy‐harvesting sectors by showing their primitive success in achieving highly efficient photovoltaic cells.^[^ ^1^ ^]^ Extensive researches in the past decade contribute to a surge of power conversion efficiency (PCE) from 4% to 25.2% (recorded in the year of 2020) at the laboratory‐perovskite‐cell.^[^ ^2^ ^]^ Although such an evolution represents a big accomplishment, the research has predominantly focused on achieving a record‐high PCE, with stability and durability often being insufficiently emphasized. Competition with incumbent photovoltaic (PV) technologies require perovskite cells to be highly stable and durable to accommodate with the industry standard of 20 year survival in outdoor conditions. Solicitation of year‐scale maintenance of a PCE floor from an initial efficiency over 18% has been released from U.S. Department of Energy (DOE).^[^ ^3^ ^]^ Advanced perovskite PV technology with both desirable PCE and durability is thus considered as a paramount step bridging the ongoing research to future commercialization.

The perovskite solar cells are thin film PV device built by layer‐by‐layer processing with incorporation of multiple functional charge extraction/transport/buffer layers and the perovskite light‐harvesting layer.^[^ ^4^, ^5^, ^6^, ^7^ ^]^ Conventionally, “n‐i‐p” structured perovskite PV device (with n‐type buffer layer being coated at the front transparent electrode and p‐type being the back contact buffer) has been widely studied, but the commonly used compact TiO_2_ front and 2,2′,7,7′‐Tetrakis[*N*,*N*‐di(4‐methoxyphenyl)amino]‐9,9′‐spirobifluorene (Spiro‐OMeTAD) back buffer layers have high‐temperature sintering concern^[^ ^8^ ^]^ and high‐cost issue,^[^ ^9^ ^]^ respectively. These would virtually rise the levelized cost of energy (LCOE) for perovskite PV products.^[^ ^10^ ^]^ Moreover, the ionically doped Spiro‐OMeTAD^[^ ^11^ ^]^ usually exhibit strong hygroscopic behavior that will accelerate the moisture‐assisted degradation of the cell.

Alternatively, inverted “p‐i‐n” structured devices (with p‐type being the front buffer and n‐type being the back contact buffer) with low‐temperature processed inorganic buffer layers have been developed aiming at a lower LCOE and higher durability. In specific, inorganic p‐type semiconductors such as NiO*_x_*,^[^ ^12^ ^]^ CuSCN,^[^ ^13^ ^]^ Cu_2_O,^[^ ^14^ ^]^ CuO,^[^ ^15^ ^]^ PbS,^[^ ^16^ ^]^ and graphene oxide (GO),^[^ ^17^, ^18^ ^]^ etc., have been introduced as front anode buffers in inverted perovskite cells. However, the back‐contact buffers lack efficient material candidates. So far, most widely used back contact electron extraction/transport materials are the fullerene‐based materials.^[^ ^16^ ^]^ While the dilemma between efficient out‐of‐plane electron transit and the in‐plane full film‐coverage represents a major challenge in those fullerene‐based top buffers (or back contact buffer).^[^ ^16^ ^]^ Replacement by inorganic metal oxide (IMO) or the IMO:fullerene composite would be a promising solution, as they could provide suitable material configuration for better‐matched energy level with different photoactive layers,^[^ ^19^ ^]^ more compact buffering to inhibit corrosion of the metal electrodes caused by the ionic diffusion (e.g., MA^+^, I^−^, etc.)^[^ ^20^ ^]^ from perovskite, and optimized electrical contact to maximize the electron extraction and transportation.^[^ ^8^ ^]^ Recently, Choy and co‐workers have developed a thick TiO_2_ backbone film as the top cathode buffer which suppressed the monomolecular Shockley−Read−Hall recombination loss and ion diffusion induced degradation.^[^ ^21^ ^]^ And various 0D n‐type IMOs (ZnO, SnO_2_, and Zn_2_SnO_4_)^[^ ^22^, ^23^, ^24^, ^25^ ^]^ have also been used as top buffering in inverted perovskite cells. Although higher durability has been observed in perovskite PV cell using these IMOs top buffering, the PCE remains compromised (≤20%^[^ ^8^, ^21^ ^]^) and inferior to their “n‐i‐p” structured counterparts (21–22%^[^ ^26^, ^27^ ^]^).

Here in this scenario, we report the utilization of 1D cation‐doped brookite TiO_2_ nanorod (NR) with embeddings of 0D fullerene as the top cathode buffering for inverted perovskite solar cell. Such a composite design was inspired by the effectiveness of 1D TiO_2_ NR embedded by 0D C_60_ on enhancement in photo‐electrochemical response in photocatalytic field.^[^ ^28^ ^]^ In solar cells, the embedded 0D fullerene (C_60_) could fill the interstitial space of the NR network, making it a more compact capping layer to protect the perovskite as well as enhance the electrical contact with the top electrode. In addition, we use the cation‐dopant of Co and Fe in TiO_2_ NR to further modify the electronic property such as energy level and conductivity, which helps improving the charge extraction and transportation at the cathode side. Overall, we employed this C_60_:M‐TiO_2_ (M = Fe or Co) composite as the top buffering layer and demonstrated the highly efficient inverted perovskite solar cell, which exhibits a high PCE over 22%. The device also exhibits 22‐fold prolonged lifetime under continuous radiation of Xenon arc lamp (AM 1.5, including UV irradiance) and a survival PCE of 21.5% after 2‐month storage in ambient atmosphere with Realtime relative humidity (RH) ranging from 20% to 87%, indicating a greatly enhanced device durability. Our attempt in TiO_2_ based 0D:1D composite opens a new dimension in simultaneously achieving highly‐efficient and ‐durable inverted perovskite PV cell, which would further inspire a broader material inventory for nanomaterials in application of electrode buffer in PV cells.

**Figure** **1**A shows the inverted “p‐i‐n” structured solar cell devices, with a 70 nm thick layer consisting of C_60_:M‐TiO_2_ composite being the top electron transfer layer (ETL). 1D TiO_2_ NRs are well‐known nanomaterials^[^ ^29^ ^]^ and have been confirmed to be superior to other dimensional nanostructures such as 0D nanoparticles, as they can offer a direct oriented path for electron so that the charge movement could have less transversal recombination loss.^[^ ^30^, ^31^ ^]^ In addition, to maximize the charge transport and electrical band structural benefits, we further dope the 1D TiO_2_ NRs with foreign elements, including cations of Fe and Co, which can tune lattice oxygen vacancy and evoke changes in electronic structure, surface potential, and other opto‐physical properties.^[^ ^32^ ^]^ Our prior study has revealed that single transition metal (M) dopants in a broad range could be homogeneously doped into monodisperse single‐crystalline brookite‐phase TiO_2_ NRs (M‐TiO_2_), which has led significant change in optoelectronic properties and made a difference in photocatalytic application.^[^ ^32^ ^]^ These homogeneously Fe‐ and Co‐ doped TiO_2_ NRs are schematized in Figure 1B‐i). The single transition metal (M) dopants does not change the 1D nanostructure or the overall TiO_2_ crystal phase. We use the transmission electron microscopy (TEM) to visualize the nanoscopic geometric feature. As shown in Figure 1B‐ii to iv, there is no physical dimensional change upon doping. Both Fe‐TiO_2_ and Co‐TiO_2_ maintain their NR feature inherited from the pristine TiO_2_. Figure 1C shows the X‐ray diffraction (XRD) spectra of different NRs. In accordance, all the NRs share the X‐ray scattering feature of brookite‐phase TiO_2_ (denoted with the lattice plane in Figure 1C). There is no significant change in lattice parameter which agrees well with the fact of low‐doping concentration and thereby the vast majority of the crystal maintains its TiO_2_ brookite‐phase. To confirm this low‐doping concentration, we employed the energy dispersive X‐ray spectroscopy (EDS) in conjunction with scanning electron microscopy (SEM) to quantify the element composition. Figure 1D compares the EDS result of Fe‐TiO_2_ and Co‐TiO_2_ nanorods where characteristic X‐ray emitted from electron bombardment with Fe and Co are detected, respectively. The quantification of chemical composition is also shown in the tables inserted in Figure 1D. 7.8 and 6.7 mol% of Co and Fe, respectively, have been verified to be incorporated within the M‐TiO_2_ NR. Thus, single transition metal doping of Co and Fe has been successfully introduced into TiO_2_ NRs.

**Figure 1:**
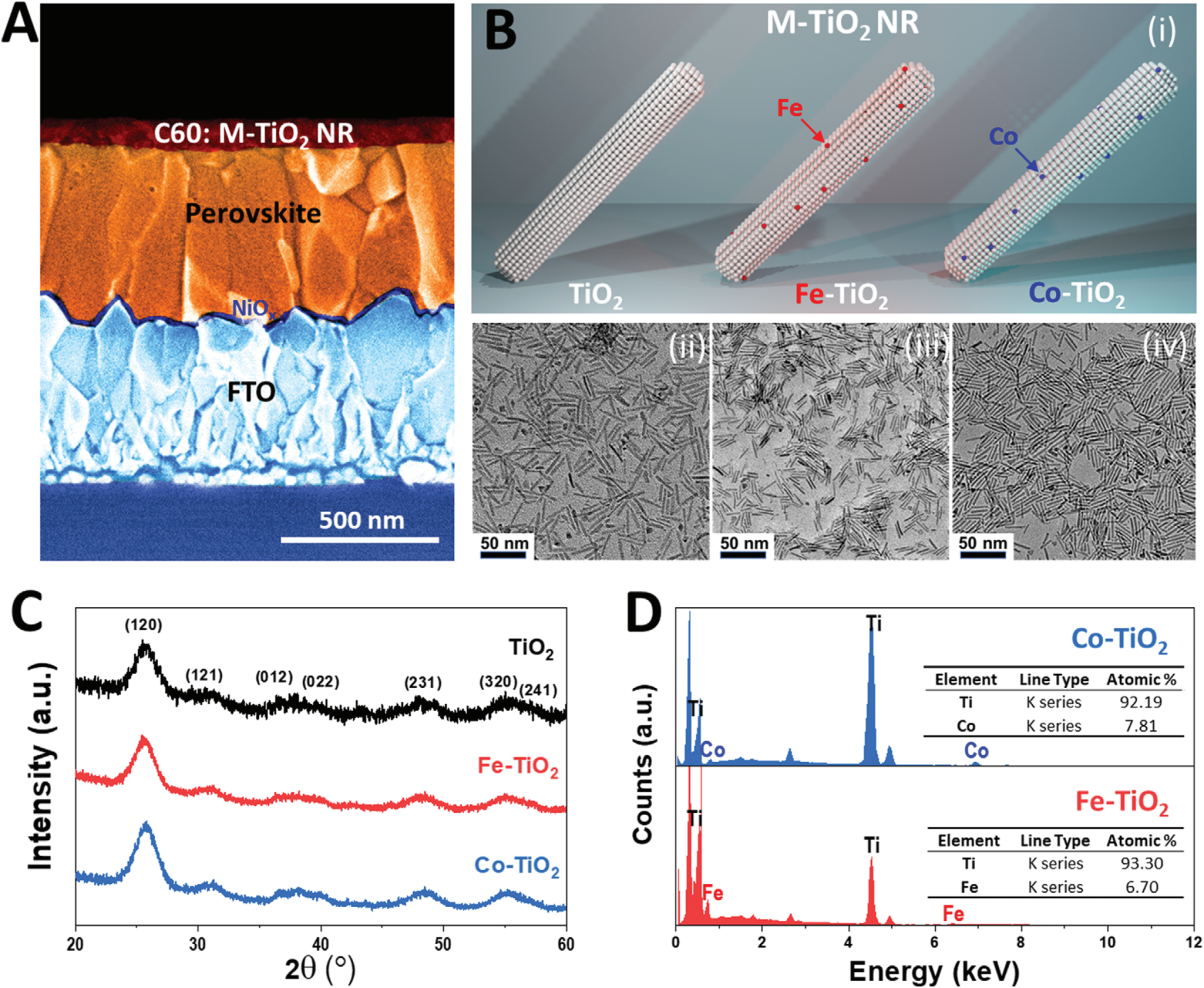
A) Cross‐sectional SEM image of FTO/NiO*_x_*/Perovskite/C_60_:M‐TiO_2_ NR composite (M = Fe or Co), showing a homogeneous layer of C_60_:M‐TiO_2_ RD as the top buffering layer for solar cell. B) Nanoscopic feature of M‐TiO_2_ NR. i) Schematic showing the pristine and single transition metal doped M‐TiO_2_. Corresponding TEM of ii) TiO_2_, iii) Fe‐TiO_2_, and iv) Co‐TiO_2_. C) XRD spectra of different M‐TiO_2_ NR, lattice planes of brookite‐phase are assigned. D) EDS results of Fe‐TiO_2_ and Co‐TiO_2_, collected from Figure S1 in the Supporting Information.

Next, we use these M‐TiO_2_ NRs as matrix for fullerene imbedding and further apply the composite as cathode buffering in inverted perovskite solar cell. The motivation of utilization of fullerene as filling in the NR matrix is to ameliorate the morphological and micro‐electrical property, as the film only consisting of NRs contains a large ratio of incontinuity and interspatial gap, which will cause severe charge scattering or trapping loss and significantly limit the charge transport.^[^ ^33^ ^]^ In this study, both fullerene and M‐TiO_2_ NRs are dissipated in nonpolar solvent such as chlorobenzene, which could be further spin‐casted on top of perovskite to form a homogeneous buffer layer of C_60_:M‐TiO_2_ composite (detailed in the Experimental Section) without degrading the bottom perovskite layer. We then compare the microscopic feature of different ETL buffer layers. **Figure** **2** displays the SEM images of different ETL buffer layers spin‐coated on top of perovskite layers. It could be seen that homogeneous ETL buffer layers with thickness of ≈70 nm have been realized using either pristine C_60_ or the C_60_:M‐TiO_2_ composite materials. We found full and homogeneous coverage by different ETL buffer layers on top of (FAPbI_3_)_0.95_(MAPbBr_3_)_0.05_ perovskite. Interestingly, it is found that the M‐TiO_2_ NR tends to have a short‐distance parallelly stacking pattern with amorphous fullerene embedded between gaps (schematized in Figure S2, Supporting Information). Such spontaneously formed parallelly stacking pattern of NR is similar to those of folded chains’ lamellae packing in polymer semi‐crystals,^[^ ^34^ ^]^ which might be due to the rearrangement of geometry in accordance to minimize the overall free energy as such a parallel packing tends to more efficiently reduce the surface energy.^[^ ^35^ ^]^ Overall, the C_60_:M‐TiO_2_ composite materials exhibit superior surface morphology and great film uniformity on top of perovskite.

**Figure 2:**
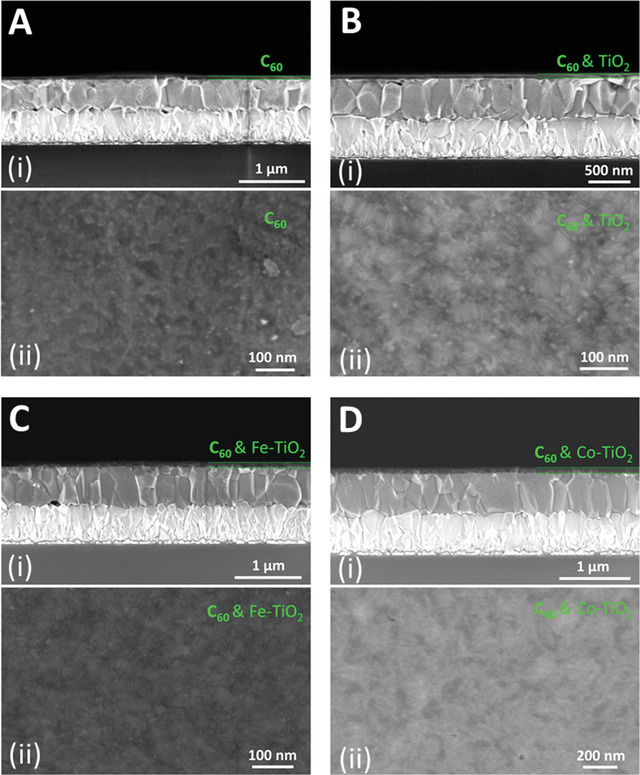
SEM images of different ETL buffer layers (on top of perovskite) consisting of A) pure C_60_, composite of B) C_60_:TiO_2_, C) C_60_:Fe‐TiO_2_ and D) C_60_:Co‐TiO_2_, where i) are the cross‐sectional SEM images and ii) are the corresponding top‐view images.

As an ETL buffer layer, efficient electron extraction ability that can facilitate photocarrier flowing from perovskite to ETL and excellent electrical properties to transport those carriers with minimized dissipation loss inside the ETL are required for high‐performance perovskite solar sell. We employ the photoluminescence (PL) study to check the charge extraction ability of different ETL buffer layers. **Figure** **3**A shows steady‐state PL spectra of perovskite itself and perovskite coated with different ETL materials (acting as quencher in the PL study). We use a constant excitation light source with identical wavelength (505 nm) and intensity for all the samples. Without any quencher, perovskite solely exhibits the highest PL intensity with a peak of ≈775 nm, a fingerprint of (FAPbI_3_)_0.95_(MAPbBr_3_)_0.05_.^[^ ^36^ ^]^ While with the presence of C_60_ quenching layer, the PL intensity exhibits significant decreased yield which is due to the charge extraction from (FAPbI_3_)_0.95_(MAPbBr_3_)_0.05_ to the C_60_ quenching layer (ETL buffer).^[^ ^11^ ^]^ Replacing the C_60_ with C_60_:M‐TiO_2_ composite materials further decreases the PL, suggesting a more efficient photocarrier quenching process at the (FAPbI_3_)_0.95_(MAPbBr_3_)_0.05_/C_60_:M‐TiO_2_ interface. We then cross‐check the results through the time‐resolved PL (TRPL) spectroscopy. Figure 3B compares the TRPL spectra of perovskite only and perovskite coated with different ETL materials. For all the bi‐layer of (FAPbI_3_)_0.95_(MAPbBr_3_)_0.05_/C_60_:M‐TiO_2_, there is a single‐exponential decay featuring the fast charge extraction across the interface. This fits well with the ideal bi‐layer model of single‐exponential photocarrier quenching derived from continuum theory,^[^ ^37^ ^]^ as some reports also display a multi‐exponential decay containing a secondary slower process (longer photocarrier lifetime) which is corresponding to the photocarrier decay inner the bulk perovskite itself other than the interface^[^ ^1^, ^38^ ^]^ (this should be excluded here). We then calculate the carrier lifetime based on following equation

(1) $$n\left(t\right)=N\;\exp\left(-\frac{t}{\tau }\right)$$

with *n*(*t*) being the PL emission photon numbers collected at time *t*, *N* being the initial photon number and *τ* being the lifetime. **Table** **1** compares the lifetime of photo‐generated carriers in perovskite after being coated with different ETL buffer layers. Compared to the perovskite itself (*τ* = 341 ns), perovskite coated by pristine C_60_ exhibits a reduced lifetime of *τ* = 138 ns; perovskite coated by C_60_:TiO_2_, C_60_:Fe‐TiO_2_ or C_60_:Co‐TiO_2_ exhibits further reduced lifetime of *τ* = 68, 54, and 41 ns, respectively. These results indicate a noticeably enhanced photocarrier quenching process in those (FAPbI_3_)_0.95_(MAPbBr_3_)_0.05_/C_60_:M‐TiO_2_ samples, where the photocarriers could be sufficiently extracted from (FAPbI_3_)_0.95_(MAPbBr_3_)_0.05_ to C_60_:M‐TiO_2_.

**Figure 3:**
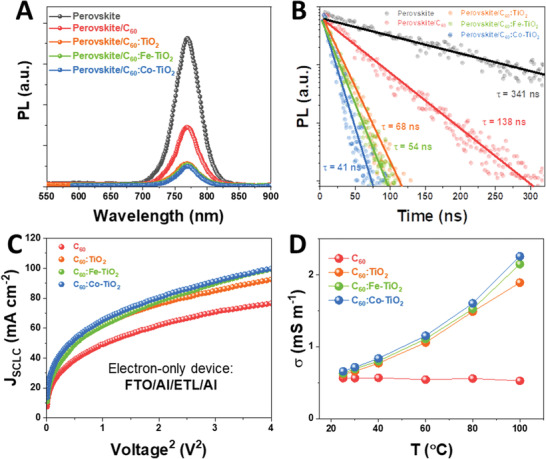
A) Steady‐state PL and B) time‐resolved PL spectra of perovskite only and perovskite coated with different ETL materials. Mobility measurement based on space‐charge‐limited current (SCLC) method: C) corresponding *J*_SCLC_‐*V* ^2^ plot of electron‐only devices with structure of FTO/Al/ETL/Al. D) The electrical conductivity measured from devices of FTO/ETL/Al at different temperatures.

**Table 1:** Photocarrier lifetime in perovskite when coated with different ETL buffer layers and electron mobility of ETL buffer layers measured from SCLC method

ETL buffer	Carrier lifetime in perovskite [ns]	Electron mobility [cm^2^ V^−1^ s^−1^]
C_60_	138	8 × 10^−5^
C_60_:TiO_2_	68	6 × 10^−4^
C_60_:Fe‐TiO_2_	54	8 × 10^−4^
C_60_:Co‐TiO_2_	41	9 × 10^−4^

As an efficient ETL buffer layer, another important role is to transfer the electron to the cathode with minimal loss, which requires the buffer material to have highly efficient electron transfer capability. We study the electrical transfer properties of these C_60_:M‐TiO_2_ ETL buffer materials. Figure 3C displays the *J*_SCLC_‐*V* ^2^ plot obtained from an electron‐only device of fluorine‐doped tin oxide (FTO)/aluminum (Al)/ETL/Al, where the *J*_SCLC_ is the dark current density of the electron‐only diode and *V* is the applied voltage. The electron mobility (*μ*) of ETL could be calculated from the equation of (using the Mott–Gurney square law):^[^ ^39^ ^]^

(2) $$J_{\mathrm{SCLC}}=\frac{9}{8}\varepsilon_{r}\varepsilon_{0}\mu \frac{V^{2}}{L^{3}}$$

with *ε*_r_ being the dielectric constant, *ε*_0_ being the permittivity of free space, *L* being the thickness of ETL, respectively. The results are shown in Table 1. The pristine C_60_ ETL displays an electron mobility of 8 × 10^−5^ cm^2^ V^−1^ s^−1^, which is comparable to prior reported values.^[^ ^40^, ^41^ ^]^ Meanwhile we found the C_60_:M‐TiO_2_ composite ETL exhibits one magnitude higher electron mobility (e.g., C_60_:Fe‐TiO_2_ displays a *μ* of 8 × 10^−4^ cm^2^ V^−1^ s^−1^), as TiO_2_ has been reported to have a higher mobility than fullerene.^[^ ^42^ ^]^ In addition, by single transition metal doping, we found the C_60_:Fe‐TiO_2_ and C_60_:Co‐TiO_2_ display slightly higher mobility than the C_60_:TiO_2_. Our prior studies have revealed that the homogeneous foreign transition metal doping in TiO_2_ could help to tune the electron band structure which has been confirmed to affect the photoelectronic behavior in photocatalytic application.^[^ ^32^ ^]^ Physically, the transition metal doping in the lattice will adjust the electronic band structure in the momentum space, while the band curvature (particularly the extreme of the band) is closely related to the effective mass of charge carrier which is inversely proportional to the carrier mobility.^[^ ^43^ ^]^ Hence the slight change in mobility of C_60_:M‐TiO_2_ composite can be ascribed to the single transition metal doping effect. To further distinguish the electrical property between pristine C_60_ ETL and C_60_:M‐TiO_2_ ETL, we measure the electrical conductivity of different ETLs at various temperatures. As shown in Figure 3D, C_60_:M‐TiO_2_ ETL exhibits slightly higher electrical conductivity (e.g., 0.66 mS m^−1^) than pristine C_60_ ETL (0.56 mS m^−1^) at room temperature (RT). As temperature increases, the C_60_:M‐TiO_2_ ETLs exhibit an increasing in conductivity (e.g., from 0.66 mS m^−1^ at 25 °C to 2.25 mS m^−1^ at 100 °C for C_60_:Co‐TiO_2_), suggesting a thermally activated conductive behavior and a higher carrier density at elevated temperatures. In contrast, electrical conductivity of the pristine C_60_ ETL slightly decreases along with the temperature's increase (shown in Figure S3 (Supporting Information), where conductivity displays a drop from 0.56 mS m^−1^ at 25 °C to 0.52 mS m^−1^ at 100 °C), presumably due to the activation of defects in C_60_ film at higher temperatures.^[^ ^44^ ^]^

Above we have shown the C_60_:M‐TiO_2_ ETL buffer layers displaying superior electron extraction and transfer properties in comparison with pristine C_60_ ETL. Additionally, for efficient perovskite solar cells, the energy level alignment between perovskite photoactive layer and the ETL is a crucial factor determining the overall device performance. We employ the Kelvin probe force microscopy (KPFM) to microscopically investigate the surface of different ETL buffer layers. **Figure** **4**A compares the topographical images of different ETLs and their CPD (corresponding contact potential difference) images. CPD reflects the potential difference between work function of tip (*Φ*_tip_) with respect to the surface potential of sample, which could be used to calculate the quasi‐Fermi‐level of electron (QFLE) of the sample by knowing the tip's work function.^[^ ^45^ ^]^ QFLE defines the population of electrons separately in the conduction band and valance band, when their population are displaced from equilibrium due to heat, light, bias, etc., which could more precisely reflect the electronic nature of a material in real case.^[^ ^43^ ^]^ Figure 4B shows the distribution plot of CPDs obtained from different samples. We use Gaussian distribution to fit the histogram of CPDs, as detailed in Note S1 (Supporting Information). It can be seen that there is an obvious difference in CPD distribution between pristine C_60_ ETL and the C_60_:M‐TiO_2_ composite ETLs. As listed in Table S1 (Supporting Information), pristine C_60_ ETL exhibits a mean value and variance of CPD of 0.54 and 0.157 respectively. While the C_60_:M‐TiO_2_ ETL exhibits higher CPD but narrower variance. For example, C_60_:Co‐TiO_2_ displays a mean value and variance of 0.98 and 0.061 respectively. The narrower CPD in those C_60_:M‐TiO_2_ ETLs suggest a higher microscopic uniformity in terms of the electronic property. We further quantify these CPDs in terms of value of QFLE in Figure 4C (detailed in Note S2 (Supporting Information) for the conversion of CPD into QFLE), along with the root‐mean‐square (RMS) roughness (*R*_q_) obtained from the corresponding topographical images. The *R*_q_ of all the ETLs display a similar value locked in a narrow range of 3.2–4.6 nm. For example, the pristine C_60_ ETL displays a *R*_q_ of 4.6 nm and C_60_:Fe‐TiO_2_ ETL displays a *R*_q_ of 4.2 nm. Taking consideration of the thickness of these ETLs (≈70 nm in Figure 2), the small *R*_q_ of <5 nm for all the C_60_:M‐TiO_2_ composite ETLs suggests a high film homogeneity which is expected to have a good electrical contact with top metal electrode. On the other hand, we found the pristine C_60_ ETL exhibits a typical QFLE of −4.53 eV (could be considered as the Realtime conduction band minimal). In contrast, C_60_:M‐TiO_2_ composite ETLs exhibit higher QFLE. Specifically, C_60_:TiO_2_, C_60_:Fe‐TiO_2_, and C_60_:Co‐TiO_2_ display the QFLE of −3.83, −4.04, and −4.09 eV, respectively.

**Figure 4:**
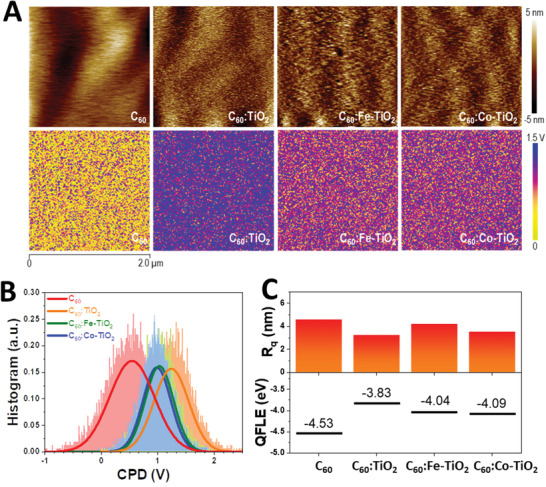
A) KPFM measurement on different ETL buffer layers. The top row shows the AFM topographies and the bottom the corresponding KPFM images. B) CPD distribution of different ETLs. C) Comparison of *R*_q_ and quasi‐Fermi‐level of electron (QFLE) of different ETLs.

These elevated QFLE in C_60_:M‐TiO_2_ ETL buffer layers are expected to have better energy level alignment with the conduction band of (FAPbI_3_)_0.95_(MAPbBr_3_)_0.05_ perovskite. **Figure** **5**A displays the diagram of energy level alignment in the inverted perovskite solar cell. At the cathode side, there is a larger energy level mismatch between the conduction band minimal (CBM) of (FAPbI_3_)_0.95_(MAPbBr_3_)_0.05_ and C_60_ (with a big difference of 530 meV). In contrast, the contact between (FAPbI_3_)_0.95_(MAPbBr_3_)_0.05_ and C_60_:TiO_2_, or C_60_:Fe‐TiO_2_ or C_60_:Co‐TiO_2_ ETLs displays smaller energy level mismatch of 170, 40, and 90 meV, respectively. This better‐matched energy level of electron secures a more efficient interfacial transfer, as verified in above PL studies. In a solar cell the photoelectrons originate from the photoexcitation of valance band (VB) electrons to CBM of perovskite layer, and they have the initial density of state (DOS) distribution at the CBM of perovskite. After that, these CBM photoelectrons will need to transfer across multiple layers/interfaces toward the electrode, through which their electrical potential will be lost. Smaller energy level offset between ETL and perovskite will reduce the potential lost during the transfer of those photoelectrons across the interfaces, leaving a higher residual potential at the electrode and thereby a higher *V*_OC_. We then use these different ETL as the top buffer layers in an inverted perovskite solar cell, with a device structure of FTO/NiO*_x_*/(FAPbI_3_)_0.95_(MAPbBr_3_)_0.05_/ETLs/BCP/Ag, where FTO is fluorine‐doped tin oxide, BCP is bathocuproine and Ag is silver. Figure 5B shows the current density–voltage (*J–V*) curve of solar cells using different ETLs. The photovoltaic parameters are listed in **Table** **2**. The inverted perovskite solar cell using pristine C_60_ ETL as top buffer exhibit a *J*_SC_ (short‐circuit current density) of 23.04 mA cm^−2^, *V*_OC_ (open‐circuit voltage) of 1.00 V, FF (fill factor) of 0.768, and PCE of 17.69%. In comparison, using C_60_:TiO_2_ ETL, we found a significant improvement in PCE of 20.89%, with simultaneously improved *J*_SC_ of 23.85 mA cm^−2^, *V*_OC_ of 1.08 V, and FF of 0.811. Moreover, using the C_60_:Fe‐TiO_2_ and C_60_:Co‐TiO_2_ ETL, there is a further improvement in device performance. In particular, the device incorporated with C_60_:Co‐TiO_2_ ETL exhibits a maximal PCE of 22.13% with *J*_SC_ of 24.38 mA cm^−2^, *V*_OC_ of 1.10 V, and FF of 0.825. We have shown above that the Co‐TiO_2_ ETL has optimized energy level alignment with (FAPbI_3_)_0.95_(MAPbBr_3_)_0.05_ perovskite (Figure 5A), higher electron extraction efficiency and superior electrical properties (Figure 3), which jointly result in the highest device efficiency in the corresponding solar cells. In particular, the higher electron mobility in those C_60_:M‐TiO_2_ ETL ensures an efficient charge transfer and thereby less charge carrier recombination losses throughout the cathode side, leading to a higher FF for the solar cell devices. The enlarged *V*_OC_ could be ascribed to the minimized energy level mismatch that reduces the potential loss during the charge transfer across the perovskite/ETL interface, as evidenced by the KPFM study.^[^ ^11^ ^]^ Such a smaller energy level offset accompanied by the more sufficient interfacial charge extraction at the perovskite/C_60_:M‐TiO_2_ ETL interface secures less potential loss.^[^ ^46^ ^]^ To verify the boosted photocurrent by the C_60_:M‐TiO_2_ ETL, we measured the incident photon‐to‐current efficiency (IPCE) spectra of inverted perovskite solar cells using different ETLs (Figure 5C). We also integrate the spectra with regarding to the solar spectra to calculate the *J*_SC_ obtained from IPCE (Figure S5, Supporting Information). The results are listed in Table 2. The *J*_SC_ obtained from IPCE of device using C_60_, C_60_:TiO_2_, C_60_:Fe‐TiO_2_, and C_60_:TiO_2_ ETL are 22.85, 23.70, 24.21, and 24.36 mA cm^−2^, respectively. These values are in great consistence to those obtained from *J–V* curves (as can be seen in Table 2) with a negligible mismatch <1%. Such a slight deviation could be related to instrumental error such as different light spectrum. Over the entire response region from 300 to 850 nm, the C_60_:M‐TiO_2_ ETL based devices exhibits overall higher IPCE intensities than the C_60_ ETL based device. Considering all the devices use the identical perovskite layer and the only difference is the ETLs, the higher IPCE then originates from the more efficient charge extraction/transport in these C_60_:M‐TiO_2_ ETLs based devices. The photocurrent hysteresis is also studied. Figure 5D displays the *J–V* curves of inverted perovskite solar cell using C_60_:Co‐TiO_2_ ETLs tested with both reverse and forward scan. The device exhibits a PCE of 21.60% under forward scan and 22.15% under reverse scan, exhibiting a relatively small hysteresis with a hysteresis index ($\frac{\mathrm{PC}E_{\mathrm{Reverse}}-\mathrm{PC}E_{\mathrm{Forward}}}{\mathrm{PC}E_{\mathrm{Reverse}}}$) of 0.025 which is at a small level comparing those reported values.^[^ ^47^ ^]^ The hysteresis in perovskite solar cell remains to be a complex phenomenon with potential origins including the chemical composition (e.g., stoichiometry, internal interfaces and trap density of perovskite), electrical history (scanning history), atmospheric conditions (e.g., light soaking, temperature, humidity, oxygen, etc.) as well as their interdependent effects. Prior study has concluded major factors dominating the photocurrent hysteresis in perovskite solar cell to be the trap‐involved charge carrier trapping/detrapping and/or the ionic motions across the interfaces and the interfacial issues are the main origins for the hysteresis.^[^ ^48^, ^49^ ^]^ Here the relatively small hysteresis might be due to the more efficient interface between the (FAPbI_3_)_0.95_(MAPbBr_3_)_0.05_ perovskite and C_60_:M‐TiO_2_ ETLs where the optimized interface contact and incorporation of the dense ETL which could block the heavy ion (Pb^2+^, I^−^) motion across the interface and relieve the hysteresis in certain degrees.^[^ ^50^ ^]^ Overall, a high efficiency over 22% has been successfully achieved in an inverted perovskite solar cell using the C_60_:Co‐TiO_2_ ETL cathode buffer. We also check the reproducibility of our devices. Figure 5E shows the statistics of PV parameters collected from 12 individual devices (Normal distribution fitting and data colony is shown in Figure S6, Supporting Information). The control device using C_60_ ETL shows an average PCE of 16.6% with a larger error range of 1.2%. While the device using C_60_:Co‐TiO_2_ ETL displays a highest average PCE of 21.4% with a smaller error of 0.8%, indicating a higher reproducibility. The higher consistence in C_60_:Co‐TiO_2_ ETL based device is much clearer in FF, where it has a range of ±1.6% while the C_60_ ETL device has a range of ±3.0%. The high consistence and reproducibility might be related to the high electronic uniformity of the ETL film (narrower CPD distribution in Figure 4B and Table S1, Supporting Information) and the ultra‐flat feature (in the cross‐sectional SEM in Figure 2). Overall, the inverted perovskite solar cell using the C_60_:M‐TiO_2_ ETL cathode buffer exhibit higher PCEs than the C_60_ ETL based device (with an average PCE of 16.6%). In addition, with Fe‐ and Co‐dopant, the average PCE could be further improved to 20.8% and 21.4%, respectively, from that of 19.9% of C_60_:TiO_2_ ETL based cell. Meanwhile, the C_60_:M‐TiO_2_ ETL based device also exhibit highest PCE over 22%, indicating the effectiveness of using this 0D:1D composite cathode buffering material.

**Figure 5:**
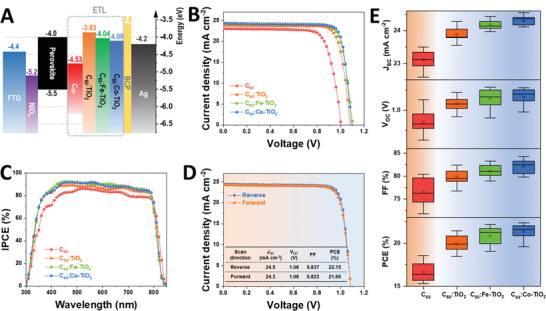
A) Diagram of energy level alignment of inverted perovskite solar cell with architecture of FTO/NiO*_x_*/perovskite/ETL buffer/BCP/Ag. B) *J–V* curve and C) IPCE spectra of inverted perovskite solar cell using different ETLs. D) *J–V* curve of inverted perovskite solar cell using C_60_:Co‐TiO_2_ ETLs. E) Statistics of photovoltaic parameters in the inverted perovskite solar cell with different ETLs.

**Table 2:** Photovoltaic parameters of inverted perovskite solar cells using different ETLs

ETLs	*J*_SC_ ^a)^	*J*_SC_ ^b)^	*V*_OC_	FF	PCE
	[mA cm^−2^]	[mA cm^−2^]	[V]		[%]
C_60_	23.04	22.85	1.00	0.768	17.69
C_60_:TiO_2_	23.85	23.70	1.08	0.811	20.89
C_60_:Fe‐TiO_2_	24.25	24.21	1.10	0.811	21.63
C_60_:Co‐TiO_2_	24.38	24.36	1.10	0.825	22.13

a) *J*_SC_ obtained from *J–V* curve

b) J_SC_ obtained from integration of IPCE spectra.

In order to understand the positive role of C_60_:TiO_2_ ETL in terms of charge carrier dynamics in the inverted device, we also carry out the transient photovoltage (TPV) and transient photocurrent (TPC) analysis. Figure S7 (Supporting Information) shows the transient measurement results of a C_60_:TiO_2_ ETL based inverted device with a comparison from a reference cell using C_60_ ETL. Briefly, we measure the photovoltage decay (TPV) under the open‐circuit condition with a pulse light for photoexcitation. During the pulse period of illumination, the separation of photo‐generated electrons and holes leads to a photovoltage generation, which decays upon light‐off due to the trap‐assisted charge carrier recombination.^[^ ^51^ ^]^ The C_60_:TiO_2_ ETL based device exhibits a slower photovoltage decay with an elongated lifetime of 5.8 µs, over twofold larger than that of 2.4 µs from the reference device (Figure S7A, Supporting Information). This observation is consistent to the enlarged *V*_OC_ in the C_60_:TiO_2_ ETL based device, both of which can be ascribed to a reduced trap density and improved extraction ability at the perovskite/C_60_:TiO_2_ ETL interface. In parallel, we also record the TPC of the same devices. Under short‐circuit condition, the photo‐generated carriers can be swept out and quickly recombined with injected carriers at corresponding electrodes and the TPC decay illustrates the transit time of photocarriers across the interfaces. The C_60_:TiO_2_ ETL based device exhibits a faster transit with a time of 206 ns, nearly threefold smaller than that of 616 ns from the reference device (Figure S7B, Supporting Information). Such an enhanced transit can be attributed to the enhanced interfacial electron extraction between the perovskite and ETL, as evidenced by the PL results. Overall, both TPV and TPC results are consistent with the conclusions from the TI/TR‐PL measurements, indicating a more efficient interfacial charge transport by using the C_60_:TiO_2_ ETL and thereby leading to reduced recombination loss and higher device performance.

Lastly, we investigate the device stability under multiple conditions. We take the inverted perovskite solar cell using the C_60_:Co‐TiO_2_ ETL cathode buffer as an example, as it displays the highest PCE. **Figure** **6**A shows the static current density and PCE as a function of the time for the C_60_:Co‐TiO_2_ ETLs devices, biased at its maximal power point (MPP) of 0.96 V and one‐sun illumination (AM 1.5) which is obtained from the power density‐bias curve in Figure S8 (Supporting Information). The C_60_:Co‐TiO_2_ ETL based cell exhibits a stabilized *J*_mp_ (current density at the maximal power point) of 23.21 mA cm^−2^ over a 300 s period. This gives rise to a steady output power of 22.28 mW cm^−2^, corresponding to a PCE of 22.28%, which is in great consistence with the value of 22.13% extracted from the *J–V* curve. In terms of long‐term stability, the perovskite device needs to have certain resistance to moisture, since the core material (halide perovskite) is sensitive to moisture.^[^ ^52^ ^]^ In solar cell device, the buffer layer on top of perovskite is even more crucial in protecting the sensitive perovskite layer underneath. While in typical “n‐i‐p” perovskite solar cells, the ionically doped Spiro‐OMeTAD is usually used as the top buffering on perovskite layer, which however exhibits strong hygroscopic feature that would accelerate the degradation of perovskite.^[^ ^11^, ^53^ ^]^ Here we use the C_60_:Co‐TiO_2_ ETL composite that exhibits a great hydrophobicity than those hygroscopic buffers in “n‐i‐p” devices. Figure 6B shows the water contact angle measurement on different ETLs directly coated on top of perovskite. It is clear that the pristine C_60_ ETL exhibits a small angle of 48°; while the composite buffer layer of C_60_:TiO_2_, C_60_:Fe‐TiO_2_, and C_60_:Co‐TiO_2_ displays increased contact angle of 73°, 84°, and 90°. Hence, utilization of these C_60_:Co‐TiO_2_ composite top buffer is anticipated to have a stronger moisture resistance in the ambient, as it would be more difficult for the moisture infiltrate into the bottom perovskite layer to trigger the degradation reaction. We then measure the working lifetime of inverted solar cells using C_60_:Co‐TiO_2_ ETL and pristine C_60_ ETL. Both devices are unencapsulated and are continuously working under an Xe lamp (AM 1.5, without any optical filter) in ambient atmosphere. It is found that the C_60_:Co‐TiO_2_ ETL based device exhibit a much slower decay than that using pristine C_60_ ETL. Briefly, the pristine C_60_ ETL device show a quick PCE drop from its original number to 0 within 14 h, while the C_60_:Co‐TiO_2_ ETL device remains 50% of its original PCE even after 110 h irradiation. We also use the linear function to fit the curve, where the slope of the fitting line represents the decay rate (ΔPCE h^−1^) of the device. The C_60_:Co‐TiO_2_ ETL device displays one‐magnitude slower decay rate (0.5% h^−1^) than the C_60_ ETL device (5.6% h^−1^), suggesting a much higher working stability. On the other hand, we also study the shelf‐life of these inverted devices (without encapsulation) using either C_60_ or C_60_:Co‐TiO_2_ ETL. All the devices are stored in ambient atmosphere with Realtime relative humidity (RH) ranging from 20–87%. The device PCE is tested once every day by the *J–V* curve measurement. Figure 6D displays the result. As expected, the control device using the pristine C_60_ ETL display a faster decay. The PCE drops from 17.1% to 14.4% in the first week and then gradually drops to 0 within one month. In contrast, the C_60_:Co‐TiO_2_ ETL device maintains an extremely excellent performance after 70 days storage, with a decent survival PCE of 21.5%. Overall, utilization of C_60_:Co‐TiO_2_ ETL as the top cathode buffer enables highly durable perovskite cells, which makes a breakthrough on the device shelf lifetime extension from a typical daily‐scale to a decent monthly‐scale. We anticipate the post encapsulation in conjunction of using our method would further prolong the device lifetime, making it possible for transition toward industry.

**Figure 6:**
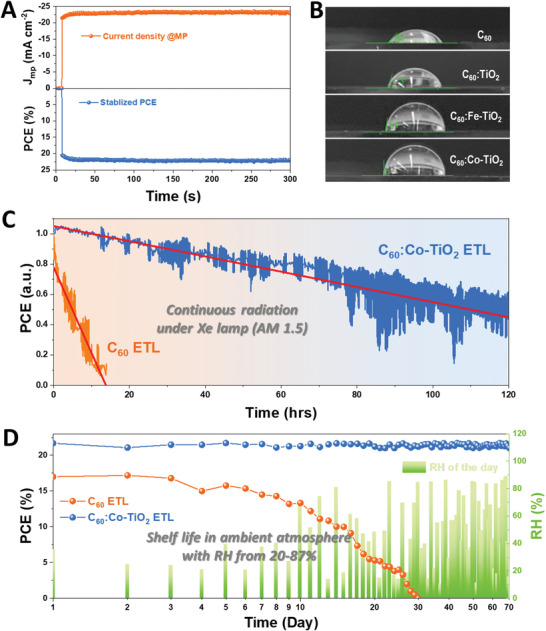
A) Static current density and PCE measured as a function of the time for the C_60_:Co‐TiO_2_ ETLs devices, biased at its maximal power point (MPP) of 0.96 V. B) Contact angle measurement of water droplet on different ETLs. C) Stability test under continuous one‐sun Xe lamp illumination in ambient air, measured by tracking the current at MPP of an unencapsulated device for 120 h. D) Device shelf life measurement by testing the *J–V* curve every day for over two months. Realtime relative humidity (RH) is also included for reference.

In summary, we have demonstrated that the utilization of 0D:1D composite consisting of fullerene and M‐TiO_2_ nanorods as the top ETL buffering in inverted perovskite solar cell represents as an efficient way to simultaneously improve the device efficiency and lifetime. Excitingly, we found the composite of C_60_:Co‐TiO_2_ ETL exhibits improved film uniformity, enhanced electron extraction and transfer ability and the better energy level matching with perovskite, as revealed by a suite of spectroscopic and optoelectronic characterizations. Moreover, we elevated the PCE baseline of inverted perovskite solar cell from <20% to ≈22% and demonstrated an extended lifetime from daily‐ to monthly scale, making it possible to accelerate the transition of this technology from lab research to the industry field. In a larger scope, the concept of 0D:1D composite buffering layer (consisting of carbon material and well‐tuned transition metal oxide nanostructures) is expected to open up more opportunities in expanding the material inventory for charge transfer layer in perovskite solar cells development and application.

## Experimental Section

##### Material

Lead (II) bromide (PbBr_2_, 99.99%), Lead (II) iodide (PbI_2_, 99.99%), and Iron (III) chloride anhydrous (FeCl_3_, 98%) were purchased from Alfa Aesar. Cobalt (II) acetylacetonate (Co(C_5_H_7_O_2_)_2_, 99%), Octadecene (ODE, 90%), and titanium chloride (TiCl_4_, 99.9%) were obtained from AcrosOrganics. Oleylamine (OAm, 70%), Oleic acid (OAc, 90%), Methylammonium bromide (MABr), Formamidinium Iodide (FAI), Ni‐(NO_3_)_2_·6H_2_O (98%), C_2_H_2_O_4_·2H_2_O (99.5%), NaOH (96%), Dimethylformanmide (DMF, extra dry, 99%), Dimethyl sulfoxide (DMSO, extra dry, 99%), 1, 2‐dichlorobenzene (DCB, extra dry, > 98%), Chlorobenzene (CB, extra dry, 99.8%), Isopropanol (extra dry, 99.8%), and other chemicals were purchased from Sigma‐Aldrich.

##### Synthesis of TiO_2_ and M‐TiO_2_ Nanorod

Brookite‐phase TiO_2_ NRs were synthesized according to the prior reported approach.^[^ ^32^ ^]^ Briefly, 10 mL OAm, 10 mL ODE and 0.48 mL OAc were first degassed under vacuum at 90 °C for 1 h to evacuate moisture and oxygen. Then the mixture was cooled down to 60 °C under nitrogen atmosphere with 1.5 mL Ti‐precursor solution containing 1.0 m OAc and 0.2 m TiCl_4_ in ODE being injected into the solution. ODE and OAc in the precursor were pre‐dried and stored in glovebox to avoid undesirable hydrolysis in TiCl_4_. The reactor was then quickly heated to 290 °C and being hold at 290 °C for 10 min. Then, another 8 mL Ti‐precursor solution was added dropwise into the reactor with a dropping rate of ≈0.3 mL min^−1^. After that, the system was cooled down to room temperature and the TiO_2_ NRs were collected and washed by addition of isopropanol, followed by centrifugation at 8000 rpm for 8 min. The product was further purified twice by the addition of isopropanol and hexane. For brookite‐phase M‐TiO_2_ NRs, the synthesis is similar to that for pristine TiO_2_ NRs, except that the TiCl_4_‐M‐oleate mixed solution was used for the M‐TiO_2_ NRs. Taking Fe‐TiO_2_ NRs as an example, the Fe‐oleate precursor was first mixed with the Ti‐precursor with a desired Ti/Fe ratio. And 1.5 mL of this mixed solution was injected into the degassed solution of 10 mL ODE, 10 mL OAm, and 0.48 mL OAc at 60 °C, which was quickly heated up to 290 °C. After 10 min heating, another 8 mL TiCl_4_‐Fe‐oleate mixed solution was introduced into the system. The as‐prepared Fe‐TiO_2_ NRs were collected and purified in the same way to that of pristine TiO_2_ NRs. The Co‐TiO_2_ NRs were synthesized in the similar method. It should be noted that this method is a general method to prepare new materials of M‐TiO_2_ NRs solely or binary doped by other transitional metals.

##### Preparation of ETL

The ETL cathode top buffers were spin‐casted from solutions containing either fullerene or NRs or mixture of fullerene and NRs. For C_60_ ETL, a solution of 10 mg mL^−1^ C_60_ in mixed solvents of CB and toluene (1:1 v/v) was spin‐casted at a rate of 1000 rpm for 50 s. For C_60_:TiO_2_ (or C_60_:M‐TiO_2_) ETLs, a solution containing 5 mg C_60_ and 5 mg TiO_2_ (or M‐TiO_2_) in 1 mL mixed solvents of CB and hexane (1:1 v/v) was spin‐casted at a rate of 1000 rpm for 50 s. All the solutions were prepared in ambient atmosphere by dispersing different materials in the solvents under ultrasonication for 30 min, followed by filtration with a 0.45 µm polyvinylidene fluoride (PVDF) filter. The solution was used right away.

##### Device Fabrication

The FTO glass was cleaned by ultrasonication in bath of detergent, deionized water, acetone and isopropanol sequentially, followed by drying in an oven at 100 °C overnight. The pre‐cleaned FTO glass substrates were then treated by UV–ozone plasma for 40 min. Afterward, a ≈20 nm thick NiO*_x_* was spin‐casted from NiO*_x_* nanocrystal solution at a spin‐rate of 4000 rpm for 40 s according to prior reports.^[^ ^54^ ^]^ Then the (FAPbI_3_)_0.95_(MAPbBr_3_)_0.05_ perovskite photoactive layer was spin‐coated by a one‐step method. Briefly, a 1.2 m mixed (FAPbI_3_)_0.95_(MAPbBr_3_)_0.05_ perovskite precursor solution with a mixed solvent of DMF:DMSO (8:2 v/v) was spin‐coated at 5000 rpm for 30 s. During the spinning process, 200 mL of CB was dropped onto the spinning surface (in the last 20 s). After that, the samples were then annealed at 70 °C for 15 min followed by annealing at 100 °C for 50 min in the glove box filled with nitrogen. After cooling down to room temperature, different ETLs were spin‐coated on top of perovskite from above solution with a spin‐rate of 1000 rpm for 50 s. Lastly, a 5 nm thick bathocuproine (BCP) and 100 nm thick silver (Ag) film was sequentially deposited on top through a shadow mask in the vacuum of <5 × 10^−6^ mbar. The device area was measured to be 0.12 cm^2^.

##### Material Characterization

Cross‐sectional SEM images of FTO/NiO*_x_*/Perovskite/C_60_:M‐TiO_2_ NR composite and SEM images of different ETL buffer layers on top of perovskite were obtained from LEO 1530 MERLIN (FESEM). The TEM images of TiO_2_ and M‐TiO_2_ NRs were obtained from FEI Spirit (120 kV). XRD spectra of TiO_2_ and M‐TiO_2_ NRs were obtained from the Philips Xpert Pro X‐ray diffractometer. Energy dispersive X‐Ray spectra (EDS) of M‐TiO_2_ NRs and their corresponding SEM images were obtained from a field emission scanning electron microscopy (FE‐SEM, FEI Sirion‐200 SEM) at an acceleration voltage of 5 kV. PL spectra of perovskite only and perovskite coated with different ETL buffer layers were obtained from a FLS1000 Photoluminescence Spectrometer, Edinburgh Instruments. KPFM images of different ETL buffer layers were obtained from Bruker Innova AFM platform.

##### Device Characterization

The solar cell device performance was characterized by measuring the *J–V* characteristics under one‐sun illumination (AM 1.5), provided by a Xenon solar simulator (Newport) in a glove‐box. The *J–V* scan rate is programed to be 100 mV s^−1^. The solar spectra were calibrated to be 100 mW cm^−2^ by a standard Si cell. The IPCE spectra were obtained from a home‐made setup consists of a stabilized Xenon lamp, beamsplitter, monochromator, photodiode connected to a power meter and a Keithley 2400 SourceMeter to record the photocurrent. Device working‐life plot was measured by tracing the photocurrent of different unencapsulated solar cells under their maximal power point voltage. All the cells are continuously working under a Xenon lamp (AM 1.5, without any optical filter) in ambient atmosphere, with a continuously supplying MMP bias for mimicking real working condition, which is different from prior “aging test” that tests periodically “working/rest” device. Device shelf‐stability was determined by measuring the *J–V* characteristics of different unencapsulated solar cells under one‐sun illumination every day. All the cells are stored in ambient atmosphere with Realtime relative humidity (RH) ranging from 20–87%. TPC and TPV measurements were conducted by a custom built‐automated setup, where a white light bias was generated from a diode array to simulate the illuminating working condition for the sample device, a pulsed laser was used as the perturbation source, and a digital oscilloscope was used to collect the data.

## Conflict of Interest

The authors declare no conflict of interest.

## Supporting information

Supporting Information

## References

[1] K. Wang , D. Yang , C. Wu , M. Sanghadasa , S. Priya , Prog. Mater. Sci. 2019, 106, 100580.

[2] F. Sahli , J. Werner , B. A. Kamino , M. Bräuninger , R. Monnard , B. Paviet‐Salomon , L. Barraud , L. Ding , J. J. Diaz Leon , D. Sacchetto , G. Cattaneo , M. Despeisse , M. Boccard , S. Nicolay , Q. Jeangros , B. Niesen , C. Ballif , Nat. Mater. 2018, 17, 820. 29891887 10.1038/s41563-018-0115-4

[3] “Funding Opportunity Announcement: Solar Energy Technologies Office Fiscal Year 2020 Funding Program | Department of Energy,” https://www.energy.gov/eere/solar/funding‐opportunity‐announcement‐solar‐energy‐technologies‐office‐fiscal‐year‐2020.

[4] N. G. Park , Mater. Today 2015, 18, 65.

[5] M. Liu , M. B. Johnston , H. J. Snaith , Nature 2013, 501, 395. 24025775 10.1038/nature12509

[6] Y. Jiang , C. Wu , L. Li , K. Wang , Z. Tao , F. Gao , W. Cheng , J. Cheng , X. Y. Zhao , S. Priya , W. Deng , Nano Energy 2018, 53, 440.

[7] K. Wang , D. Yang , C. Wu , J. Shapter , S. Priya , Joule 2019, 3, 311.

[8] D. Yang , X. Zhang , K. Wang , C. Wu , R. Yang , Y. Hou , Y. Jiang , S. Liu , S. Priya , Nano Lett. 2019, 19, 3313. 30986075 10.1021/acs.nanolett.9b00936

[9] H. D. Pham , Z. Wu , L. K. Ono , S. Manzhos , K. Feron , N. Motta , Y. Qi , P. Sonar , Adv. Electron. Mater. 2017, 3, 1700139.

[10] Z. Song , C. L. McElvany , A. B. Phillips , I. Celik , P. W. Krantz , S. C. Watthage , G. K. Liyanage , D. Apul , M. J. Heben , Energy Environ. Sci. 2017, 10, 1297.

[11] K. Wang , X. Liu , R. Huang , C. Wu , D. Yang , X. Hu , X. Jiang , J. C. Duchamp , H. Dorn , S. Priya , ACS Energy Lett. 2019, 4, 1852.

[12] X. W. Hu , X.‐F. Jiang , X. B. Xing , L. Nian , X. Y. Liu , R. Huang , K. Wang , H.‐L. Yip , G. F. Zhou , Sol. RRL 2018, 2, 1800083.

[13] I. S. Jin , J. H. Lee , Y. W. Noh , S. H. Park , J. W. Jung , Inorg. Chem. Front. 2019, 6, 2158.

[14] A. Savva , I. T. Papadas , D. Tsikritzis , A. Ioakeimidis , F. Galatopoulos , K. Kapnisis , R. Fuhrer , B. Hartmeier , M. F. Oszajca , N. A. Luechinger , S. Kennou , G. S. Armatas , S. A. Choulis , ACS Appl. Energy Mater. 2019, 2, 2276. 31168522 10.1021/acsaem.9b00070 PMC6543770

[15] W. Sun , Y. Li , S. Ye , H. Rao , W. Yan , H. Peng , Y. Li , Z. Liu , S. Wang , Z. Chen , L. Xiao , Z. Bian , C. Huang , Nanoscale 2016, 8, 10806. 27167080 10.1039/c6nr01927g

[16] T. Liu , K. Chen , Q. Hu , R. Zhu , Q. Gong , Adv. Energy Mater. 2016, 6, 1600457.

[17] E. Nouri , M. R. Mohammadi , P. Lianos , Chem. Commun. 2017, 53, 1630. 10.1039/c6cc09876b 28097274

[18] T. H. Chowdhury , M. Akhtaruzzaman , M. E. Kayesh , R. Kaneko , T. Noda , J. J. Lee , A. Islam , Sol. Energy 2018, 171, 652.

[19] N. J. Jeon , H. Na , E. H. Jung , T. Y. Yang , Y. G. Lee , G. Kim , H. W. Shin , S. Il Seok , J. Lee , J. Seo , Nat. Energy 2018, 3, 682.

[20] E. Bi , H. Chen , F. Xie , Y. Wu , W. Chen , Y. Su , A. Islam , M. Grätzel , X. Yang , L. Han , Nat. Commun. 2017, 8, 1. 28604673 10.1038/ncomms15330 PMC5472713

[21] Y. Zhao , H. Zhang , X. Ren , H. L. Zhu , Z. Huang , F. Ye , D. Ouyang , K. W. Cheah , A. K. Y. Jen , W. C. H. Choy , ACS Energy Lett. 2018, 3, 2891.

[22] J. You , L. Meng , T. Bin Song , T. F. Guo , W. H. Chang , Z. Hong , H. Chen , H. Zhou , Q. Chen , Y. Liu , N. De Marco , Y. Yang , Nat. Nanotechnol. 2016, 11, 75. 26457966 10.1038/nnano.2015.230

[23] Z. Zhu , Y. Bai , X. Liu , C. C. Chueh , S. Yang , A. K. Y. Jen , Adv. Mater. 2016, 28, 6478. 27168338 10.1002/adma.201600619

[24] X. Liu , C. C. Chueh , Z. Zhu , S. B. Jo , Y. Sun , A. K. Y. Jen , J. Mater. Chem. A 2016, 4, 15294.

[25] N. Shibayama , H. Kanda , S. Ichi Yusa , S. Fukumoto , A. K. Baranwal , H. Segawa , T. Miyasaka , S. Ito , Nano Convergence 2017, 4, 18. 28804699 10.1186/s40580-017-0113-2 PMC5532399

[26] K. Wang , C. Wu , Y. Hou , D. Yang , W. Li , G. Deng , Y. Jiang , S. Priya , Joule 2020, 4, 615.

[27] P. Zhu , S. Gu , X. Luo , Y. Gao , S. Li , J. Zhu , H. Tan , Adv. Energy Mater. 2020, 10, 1903083.

[28] Y. Z. Long , Y. Lu , Y. Huang , Y. C. Peng , Y. J. Lu , S. Z. Kang , J. Mu , J. Phys. Chem. C 2009, 113, 13899.

[29] C. Wu , K. Wang , M. Batmunkh , A. S. R. Bati , D. Yang , Y. Jiang , Y. Hou , J. G. Shapter , S. Priya , Nano Energy 2020, 70, 104480.

[30] L. C. Kao , C. J. Lin , C. L. Dong , C. L. Chen , S. Y. H. Liou , Chem. Commun. 2015, 51, 6361. 10.1039/c5cc00287g 25761526

[31] X. Q. Gong , A. Selloni , M. Batzill , U. Diebold , Nat. Mater. 2006, 5, 665. 16845415 10.1038/nmat1695

[32] Z. Zhang , Q. Wu , G. Johnson , Y. Ye , X. Li , N. Li , M. Cui , J. D. Lee , C. Liu , S. Zhao , S. Li , A. Orlov , C. B. Murray , X. Zhang , T. B. Gunnoe , D. Su , S. Zhang , J. Am. Chem. Soc. 2019, 141, 16548. 31535853 10.1021/jacs.9b06389

[33] A. A. Navab , A. Nemati , A. A. Navab , H. M. M. Abad , in AIP Conf. Proc., American Institute Of Physics Inc., New York 2018, p. 020015.

[34] Y. Li , Z. Wang , T. He , Crystals 2017, 7, 115.

[35] T. Wang , J. Zhuang , J. Lynch , O. Chen , Z. Wang , X. Wang , D. LaMontagne , H. Wu , Z. Wang , Y. C. Cao , Science 2012, 338, 358. 23087242 10.1126/science.1224221

[36] Y. Lv , B. Cai , Q. Ma , Z. Wang , J. Liu , W. H. Zhang , RSC Adv. 2018, 8, 20982. 10.1039/c8ra03559h PMC9080882 35542345

[37] E. M. Y. Lee , W. A. Tisdale , J. Phys. Chem. C 2015, 119, 9005. 10.1021/acs.jpcb.5b01886 26106811

[38] A. Bercegol , F. J. Ramos , A. Rebai , T. Guillemot , D. Ory , J. Rousset , L. Lombez , J. Phys. Chem. C 2018, 122, 24570.

[39] J. W. Jung , W. H. Jo , Adv. Funct. Mater. 2010, 20, 2355.

[40] N. Y. Doumon , M. V. Dryzhov , F. V. Houard , V. M. Le Corre , A. Rahimi Chatri , P. Christodoulis , L. J. A. Koster , ACS Appl. Mater. Interfaces 2019, 11, 8310. 30701959 10.1021/acsami.8b20493 PMC6396122

[41] Y. Zhang , I. Murtaza , H. Meng , J. Mater. Chem. C 2018, 6, 3514.

[42] J. Peng , T. Duong , X. Zhou , H. Shen , Y. Wu , H. K. Mulmudi , Y. Wan , D. Zhong , J. Li , T. Tsuzuki , K. J. Weber , K. R. Catchpole , T. P. White , Adv. Energy Mater. 2017, 7, 1601768.

[43] R. A. Serway , C. J. Moses , C. A. Moyer , Modern Physics, Cengage Learning, Boston 2004.

[44] A. Belu‐Marian , R. Manaila , T. Stoica , A. Dragomir , M. Manciu , A. Devenyi , T. Braun , Fullerene Sci. Technol. 1995, 3, 495.

[45] Y. Hou , K. Wang , D. Yang , Y. Jiang , N. Yennawar , K. Wang , M. Sanghadasa , C. Wu , S. Priya , ACS Energy Lett. 2019, 4, 2646.

[46] S. Mahesh , J. M. Ball , R. D. J. Oliver , D. P. McMeekin , P. K. Nayak , M. B. Johnston , H. J. Snaith , Energy Environ. Sci. 2020, 13, 258.

[47] R. Wang , J. Xue , L. Meng , J. Lee , Z. Zhao , P. Sun , L. Cai , T. Huang , Z. Wang , Z.‐K. Wang , Y. Duan , J. L. Yang , S. Tan , Y. Yuan , Y. Huang , Y. Yang , Joule 2019, 3, 1464.

[48] J. H. Heo , H. J. Han , D. Kim , T. K. Ahn , S. H. Im , Energy Environ. Sci. 2015, 8, 1602.

[49] K. Wang , L. Zheng , T. Zhu , L. Liu , M. L. Becker , X. Gong , Nano Energy 2020, 67, 104229.

[50] Y. Zhang , M. Liu , G. E. Eperon , T. C. Leijtens , D. McMeekin , M. Saliba , W. Zhang , M. De Bastiani , A. Petrozza , L. M. Herz , M. B. Johnston , H. Lin , H. J. Snaith , Mater. Horiz. 2015, 2, 315.

[51] L. Zuo , H. Guo , D. W. deQuilettes , S. Jariwala , N. De Marco , S. Dong , R. DeBlock , D. S. Ginger , B. Dunn , M. Wang , Y. Yang , Sci. Adv. 2017, 3, 1700106. 10.1126/sciadv.1700106 PMC5567759 28845446

[52] K. Wang , D. Yang , C. Wu , J. Shapter , S. Priya , Joule 2019, 3, 311.

[53] C. Wu , K. Wang , X. Feng , Y. Jiang , D. Yang , Y. Hou , Y. Yan , M. Sanghadasa , S. Priya , Nano Lett. 2019, 19, 1251. 30694687 10.1021/acs.nanolett.8b04778

[54] F. Jiang , W. C. H. Choy , X. C. Li , D. Zhang , J. Q. Cheng , Adv. Mater. 2015, 27, 2930. 25820687 10.1002/adma.201405391

