# Supplementary material for: 22% Efficiency Inverted Perovskite Photovoltaic Cell Using Cation‐Doped Brookite TiO2 Top Buffer
Source: Adv Sci (Weinh). 2020 Jul 2;7(16):2001285. doi: 10.1002/advs.202001285 (PMC7435259; doi:10.1002/advs.202001285)
Supplement: Supplementary file 1 — Supporting Information [file ADVS-7-2001285-s001.pdf]

Support Information
For

22% Efficiency Inverted Perovskite Photovoltaic Cell using Cation-Doped Brookite TiO_2 Top Buffer

Xiaowen Hu,[1,2#]* Chang Liu,[3#] Zhiyong Zhang,[3] Xiao-fang Jiang,[1]* Juan Garcia,[3] Colton Sheehan,[3] Lingling Shui,[1] Shashank Priya,[4] Guofu Zhou,[1,2]*, Sen Zhang,[3]* Kai Wang[4]*

[1]Guangdong Provincial Key Laboratory of Optical Information Materials and Technology & Institute of Electronic Paper Displays, South China Academy of Advanced Optoelectronics, South China Normal University, Guangzhou 510006, China
[2]SCNU-TUE Joint Lab of Device Integrated Responsive Materials (DIRM), National Center for International Research on Green Optoelectronics, South China Normal University, Guangzhou 510006, China
[3]Department of Chemistry, University of Virginia, Charlottesville, Virginia 22904, United States
[4]Material Research Institute, Pennsylvania State University, University Park, PA 16802, USA

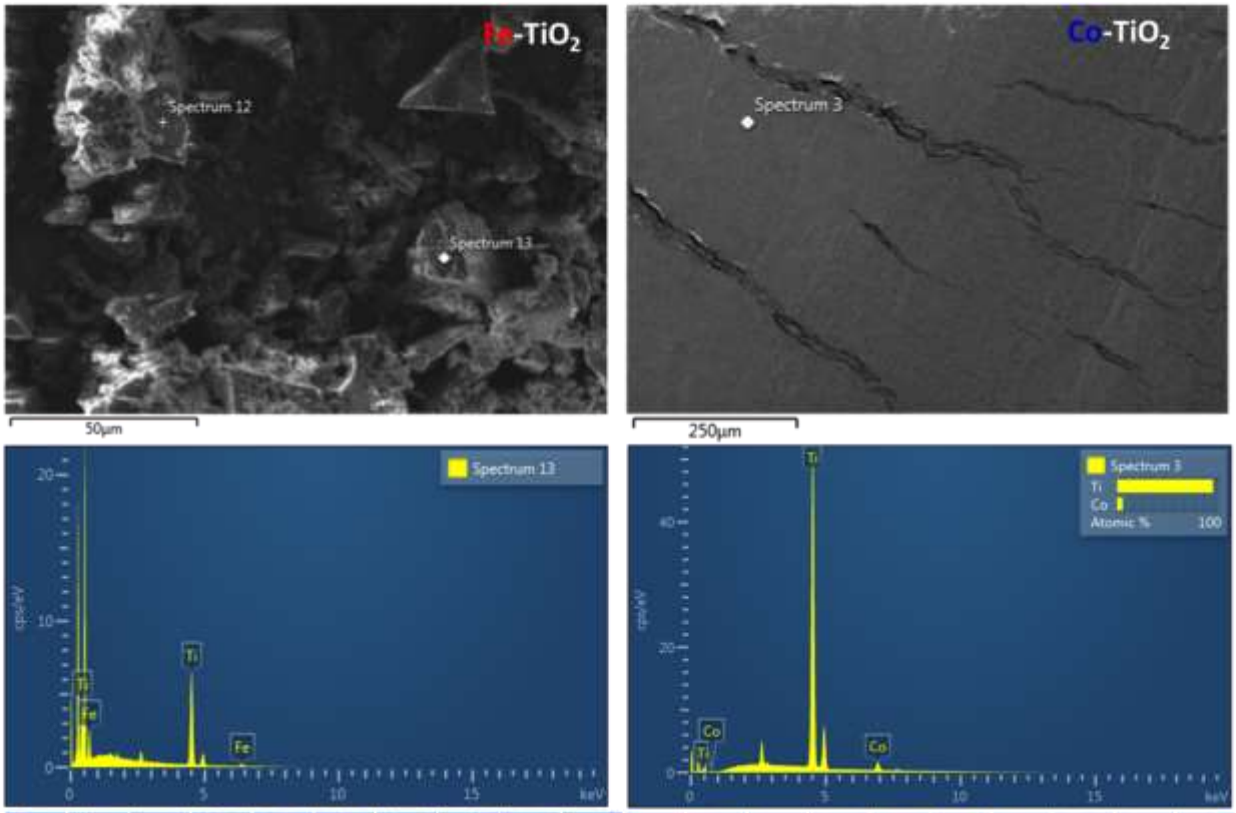

Element	Line Type	Apparent Concentration	k Ratio	Wt%	Wt% Sigma	Atomic %	Standard Label	Factory Standard	Standard Calibration Date
Ti	K series	25.12	0.25124	92.27	1.09	93.30	Ti	Yes	
Fe	K series	2.00	0.01995	7.73	1.09	6.70	Fe	Yes	
Total:				100.00		100.00			

Element	Line Type	Apparent Concentration	k Ratio	Wt%	Wt% Sigma	Atomic %	Standard Label	Factory Standard	Standard Calibration Date
Ti	K series	85.15	0.85150	90.56	0.07	92.19	Ti	Yes	
Co	K series	8.07	0.08068	9.44	0.07	7.81	Co	Yes	
Total:				100.00		100.00			

Fig. S1 SEM and corresponding EDS spectra of Fe-TiO_2 and Co-TiO_2. The molar ratio of dopant is determined from the EDS result. Both Fe and Co are recognized in the TiO_2 nanorods (NRs).

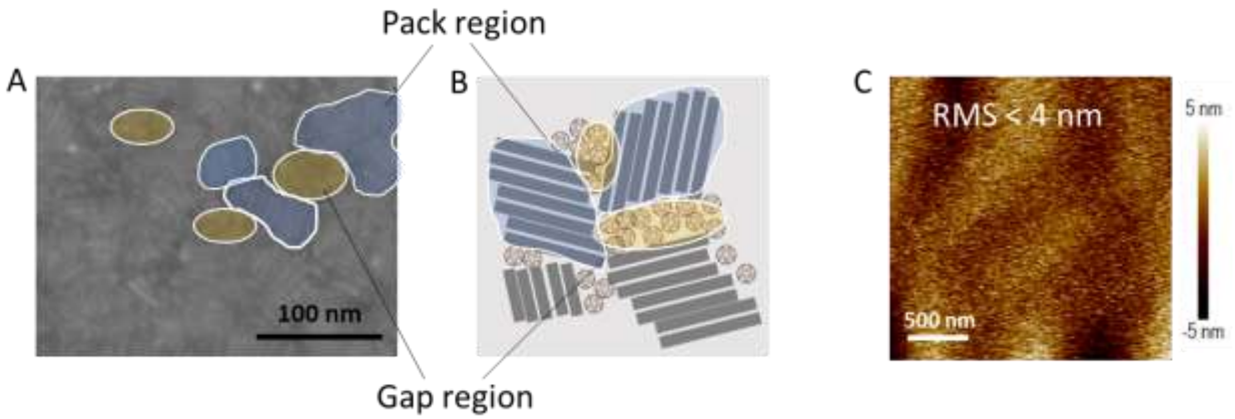

Fig. S2 (A) SEM images of nanocomposite layer of C_{60}:TiO_2 and **(B)** the schematic illustrations of the nanomorphology. The 1D NRs displays a short-distant ordering (paralleled packing, denoted as the 'pack region') feature where the 0D C_{60} could fill in the gaps (denoted as the 'gap region') in the 1D NRs packing network. We further incorporate **(C)** an AFM topography image of the nanocomposite, where a small here roughness < 4 nm demonstrates a smoothened surface which is due to the gap filling effect by the fullerene, otherwise a pristine TiO_2 NDs network would have a large surface roughness.

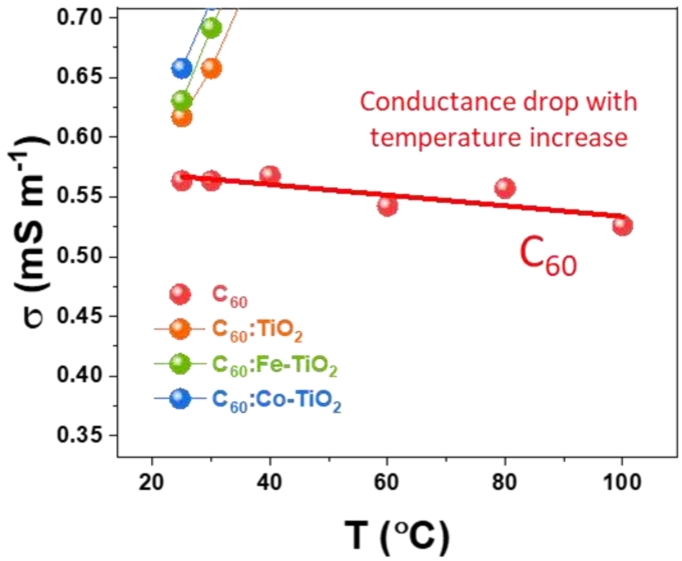

Fig. S3 Electrical conductivity dependence on temperature for the pristine C_{60} ETL, which shows a slight decrease along with the temperature's increase.

SI Note. 1 CPD Gaussian Analysis

To quantify the CPD results, we utilized the Gaussian analysis to give an in-depth inspection. The Gaussian equation is used to fit the statistical data:[1]

$$g(x) = \frac{1}{\sigma\sqrt{2\pi}} e^{-\frac{1}{2}(\frac{x-\mu}{\sigma})^2} \qquad \text{(S1)}$$

with σ being the standard deviation and μ the mean value. **Fig. S4** shows the CPD results from this study and mathematical interpretation of one Gaussian curve. We utilized the mathematical expression of to calculate deviation and mean. **Table S1** shows the results. It is clear that the C_{60} exhibits the largest variance ($\sigma^2 = w^2$)while the C_{60}:Fe-TiO_2 ETL displays the smallest variance. A smaller variance means a higher electronic uniformness.

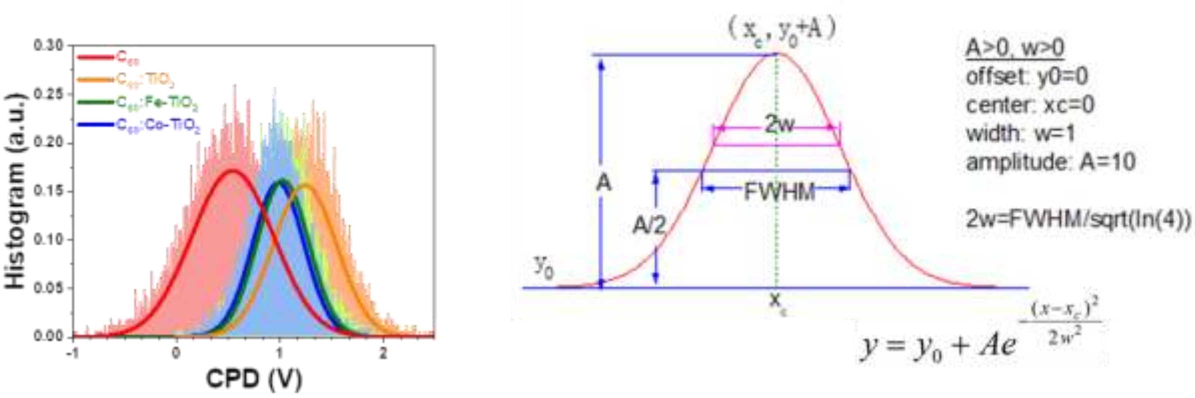

Fig. S4 CPD distribution and corresponding Gaussian analysis.

Table S1 List of mean value ($\mu = x_c$) and variance ($\sigma^2 = w^2$) of CPD for different ETLs.

ETLs	μ	σ^2
C_{60}	0.54	0.157
C_{60}:TiO_2	1.24	0.095
C_{60}:Fe-TiO_2	1.03	0.063
C_{60}:Co-TiO_2	0.98	0.061

SI Note. 2 Conversion of CPD to QFLE

QFLE of different ETLs can be calculated based on the contact potential difference between tip and sample surface measured in KPFM characterization:[2]

$$CPD = \varphi_{tip} - \varphi_{sample} \qquad \text{(S2)}$$

with φ_{tip} and φ_{sample} being the work function of KPFM tip and the sample surface. φ_{sample} is corresponding to the quasi-fermi level of electrons (QFLE). φ_{tip} can be calibrated by measuring the CPD with standard highly oriented pyrolytic graphite (HOPG), which has a work function of 4.6 eV.[3] Therefore, the QFLE of ETLs can be simplified into the equation of:

$$QFLE = e[-4.6 + (CPD_{ETL} - CPD_{HOPG})] \qquad \text{(S3)}$$

with e being the elementary charge, CPD_{ETL} and CPD_{HOPG} is the measured contact potential difference of KPFM tip with regarding to ETL and standard HOPG, respectively. Based on the CPD values, we calculated the QFLE of different ETLs in **Figure 4C**.

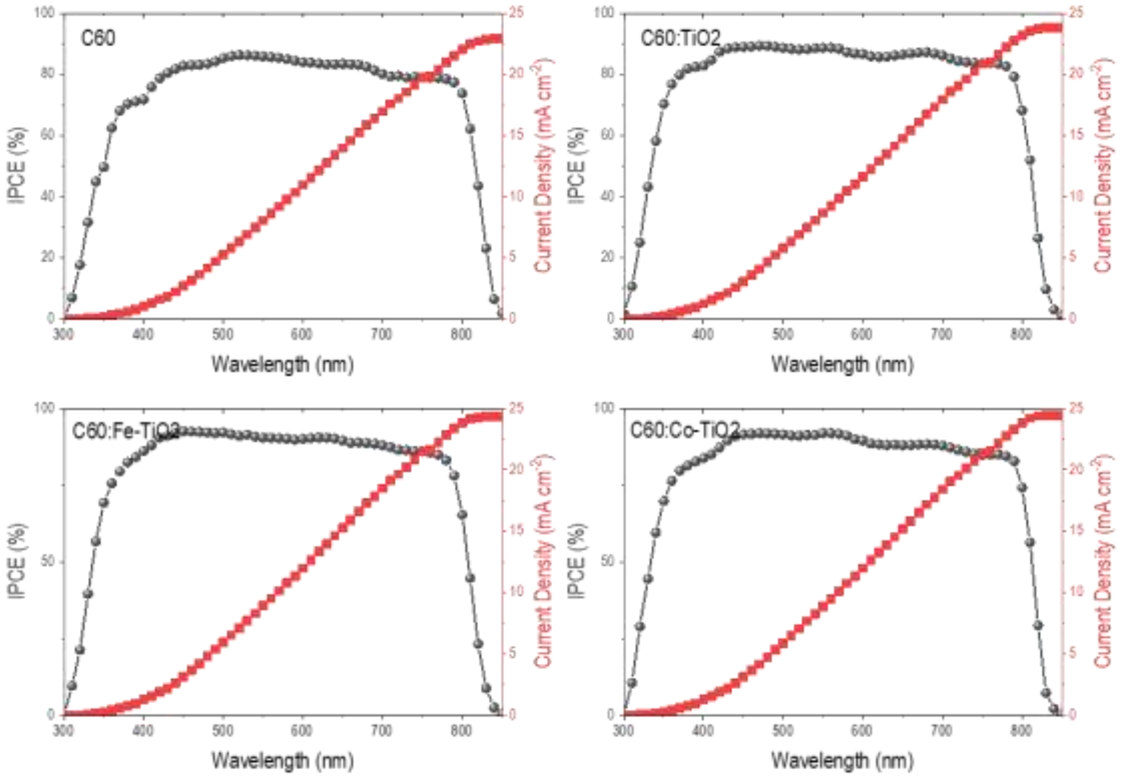

Fig. S5 IPCE spectra and corresponding current integration of inverted perovskite solar cells using different ETL materials.

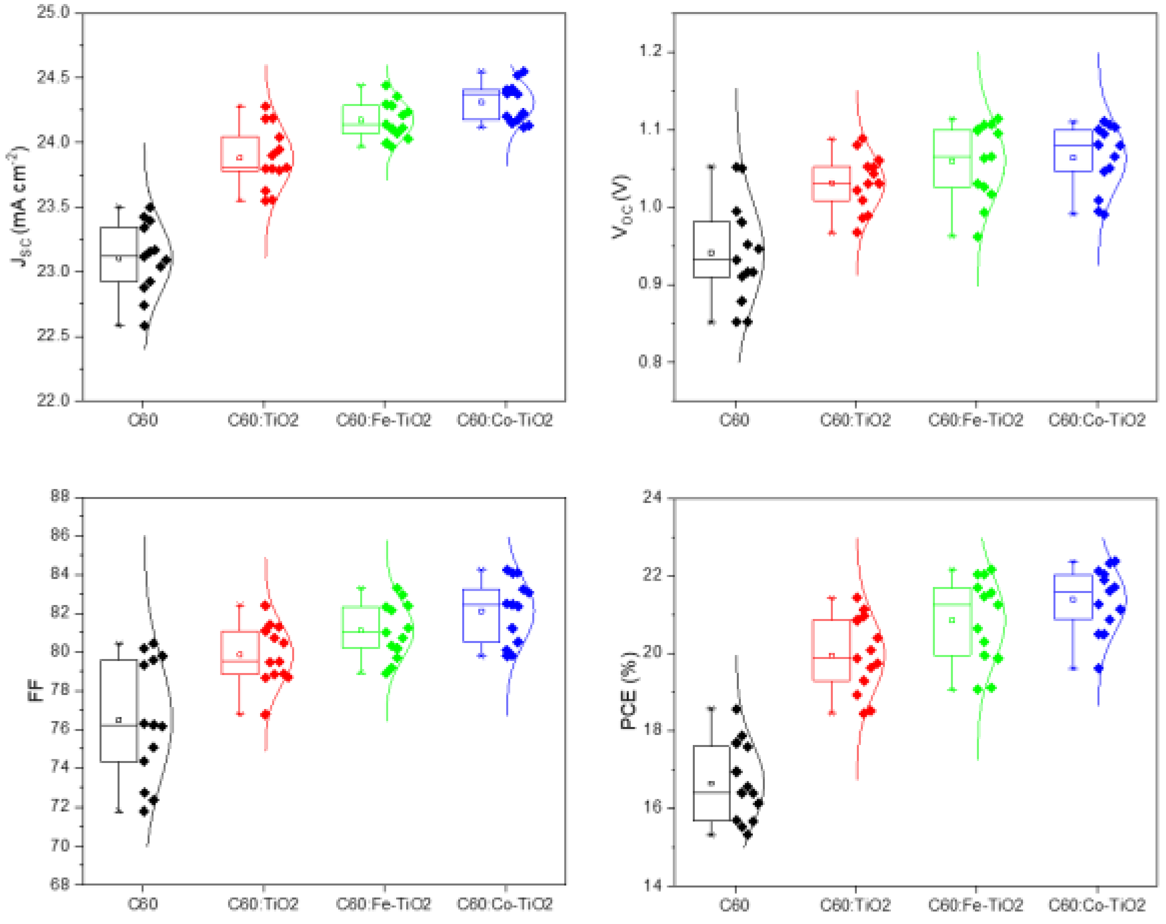

Fig. S6 Statistics and Normal fitting of inverted perovskite PV cell parameters with regarding to different ETLs.

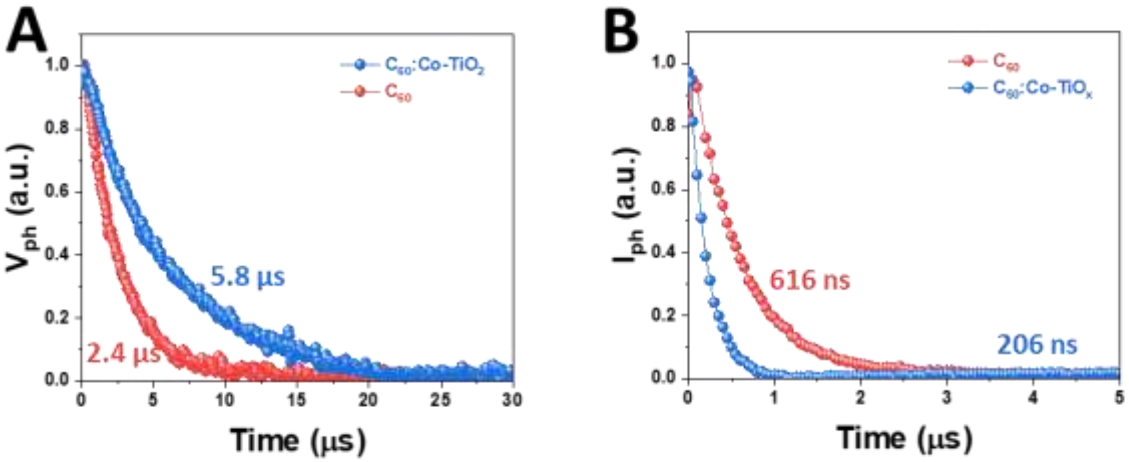

Fig. S7 Transient photovoltage (TPV) and photocurrent (TPC) study. **(A)** TPV and **(B)** TPC measurements of inverted perovskite solar cells using C_{60} and C_{60}:TiO_2 ETLs.

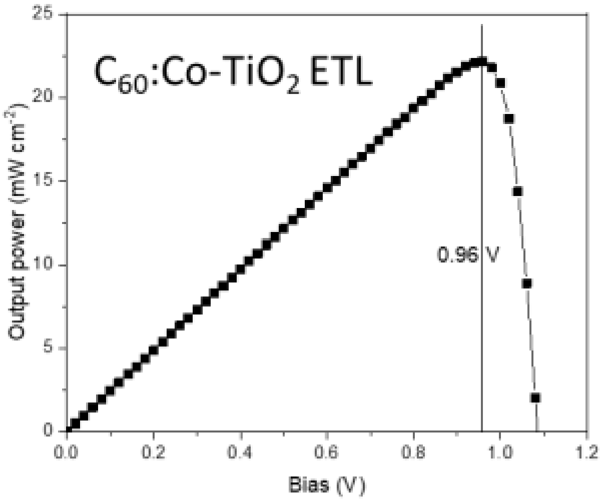

Fig. S8 Output power-bias plot for inverted perovskite PV cell using C_{60}:Co-TiO_2 ETL. The maximal power point is located at a bias supplied with 0.96 V.

SI Reference

[1] G. L. Squires, *Practical Physics*, Cambridge University Press, **2001**.

[2] W. Melitz, J. Shen, S. Lee, J. S. Lee, A. C. Kummel, R. Droopad, E. T. Yu, *J. Appl. Phys.* **2010**, *108*, 023711.

[3] D. Yang, R. Yang, K. Wang, C. Wu, X. Zhu, J. Feng, X. Ren, G. Fang, S. Priya, S. (Frank) Liu, *Nat. Commun.* **2018**, *9*, 1.
